# Neurobiological Consequences of High-Fat High-Sugar Diets on the Mesocorticolimbic System: a Narrative Review

**DOI:** 10.1007/s13668-026-00729-5

**Published:** 2026-01-16

**Authors:** Aslıhan Atar

**Affiliations:** 1 Department of Nutrition and Dietetics, Faculty of Health Science, Istanbul Beykent University, Istanbul, Turkey; 2 Department of Nutrition and Dietetics, Institute of Health Sciences, Istanbul Medipol University, Istanbul, Turkey

**Keywords:** High fat high sugar diet, Mesocorticolimbic system, Food addiction, Neuroplasticity

## Abstract

**Purpose of Review:**

This narrative review aims to examine the neurobiological consequences of high-fat, high-sugar (HFHS) diets on the mesocorticolimbic reward system. Emphasis is placed on how dopamine and opioid signaling interact to drive maladaptive behaviors such as compulsive eating and food addiction.

**Recent Findings:**

High-fat, high-sugar (HFHS) diets have a profound impact on the mesocorticolimbic reward system, altering the function of both dopamine and opioid signaling. Evidence from animal and human studies shows that acute consumption of HFHS foods produces supra-additive effects, boosting dopamine release in the Ventral Tegmental Area-Nucleus Accumens (VTA-NAc) pathway and enhancing pleasure through µ-opioid receptor activation, which reinforces repeated intake. Chronic exposure, however, results in maladaptive neuroplasticity, including downregulation of D2 receptors, weakened dopamine signaling, synaptic desensitization, and structural impairments in the prefrontal cortex. These changes parallel neural adaptations observed in substance use disorders, manifesting as tolerance, loss of control, and cue-induced craving. The opioid system also contributes to stress-related comfort eating. In contrast, individual variability in response to treatments such as the opioid antagonist naltrexone has been linked to genetic factors, including Opioid Receptor Mu 1 (OPRM1) polymorphisms.

**Summary:**

HFHS diets profoundly reshape the brain’s reward circuitry, promoting tolerance, craving, and compulsive consumption that mirror substance addiction. These findings support the conceptualization of food addiction as a neurobiological condition and highlight the importance of personalized treatment approaches. A better understanding of dopaminergic and opioid system interactions will inform targeted interventions to prevent and manage diet-related obesity and eating disorders.

## Introduction

Over the last half-century, changes in industrial food production have profoundly influenced dietary habits, steering people away from traditional, natural eating patterns and toward a growing dependence on ultra-processed foods [1]. These foods are generally high in energy density but low in nutritional value. Ultra-processed foods are characterized by high levels of refined carbohydrates and additives, as well as significant concentrations of polyunsaturated fatty acids, particularly n-6 linoleic acid [2]. This fatty acid is a primary lipid component in many industrial seed oils utilized in food processing. Linoleic acid is highly prone to oxidation during processing and storage, leading to the formation of bioactive lipid oxidation products. Recent studies show that consuming excessive amounts of oxidized n-6 fatty acids can disrupt metabolic signaling, increase inflammation, and impair the brain’s control of food intake [3]. This may also affect reward-driven eating behavior.

In this context, it has been suggested that today’s eating habits are not only responsible for the increase in metabolic diseases such as obesity and type 2 diabetes, but also for the spread of neuropsychiatric problems such as poor cognitive control, impulsivity, and compulsive eating [4, 5]. Excessive dopaminergic stimulation caused by foods high in fat and glycemic load is considered one of the underlying mechanisms of these behavioral tendencies [6].

The behavioral and neurochemical similarities between substance use disorders and overeating were the basis for the original formulation of the term “food addiction” [7]. According to this viewpoint, eating can transcend a homeostatic need and develop into a hedonistic phenomenon characterized by impulsivity [5, 8]. According to functional neuroimaging research and clinical observations, some people experience addiction-like reactions to extremely appetizing foods, especially those high in fat and sugar [9]. In these situations, people are predisposed to compulsive patterns of consumption due to a combination of hypersensitivity of the reward circuitry and a lack of cognitive control [4].

The mesolimbic dopamine pathway is a key part of this framework. The functional connectivity among the ventral tegmental area (VTA), nucleus accumbens (NAc), and prefrontal cortex (PFC) regulates the processes of reward anticipation, motivational drive, and reinforcement learning [10]. Prolonged overstimulation of this system may lead to neuroplastic changes that result in tolerance, desensitization, and loss of control characteristics of addictive behavior.

The main goal of this review is to look at how high-fat, high-sugar (HFHS) diets affect the brain’s reward system on a neurobiological level, with a focus on how dopamine and opioid signaling lead to destructive behavior. Using recent research to identify possible neurobiological targets for therapeutic intervention, a secondary goal is to assess how HFHS diets contribute to the pathophysiology of eating disorders and food addiction.

## Main Structures of the Mesocorticolimbic Circuit

The mesocorticolimbic dopamine pathway is central to the reward system, controlling both motivational and reinforcement-based actions. This circuit is made up of three primary structures: the VTA, the NAc, and the PFC [11]. The VTA is the main region in the rostral section of the mesencephalon that contains the cell bodies of most dopamine neurons. The VTA links the reward circuit using mesolimbic and mesocortical pathways. It sends dopamine signals to parts of the limbic system, like the nucleus accumbens, amygdala, and hippocampus, which help control motivation, emotion, and memory. It also connects to the prefrontal cortex, which is important for executive control and decision-making [11]. These projections are crucial for both the creation of learnt reward expectations and the maintenance of goal-directed behaviors.

The NAc, located in the ventral striatum, serves as a hub that translates motivational signals into behavior. By processing dopaminergic input especially through D1 receptors it helps shape appropriate responses and influences how strongly external rewards are experienced. In this case, the NAc serves as the neuronal substrate of the “wanting” system, processing the motivational drive to obtain the reward rather than evaluating its actual value [12, 13].

The third key component of the circuit is the PFC, the highest-level cognitive region in charge of regulating executive functions. The dorsolateral PFC is crucial for attention regulation, goal-oriented actions, and working memory, whereas the orbitofrontal cortex (OFC) is essential for assessing reward value and evaluating reward-punishment outcomes [14]. Dopaminergic input regulates both areas, harmonizing impulsive decision-making with behavioral regulation. Structural or functional abnormalities in the PFC can result in clinical problems such as excessive eating, addictive behavior, and loss of cognitive control.

**Fig. 1:**
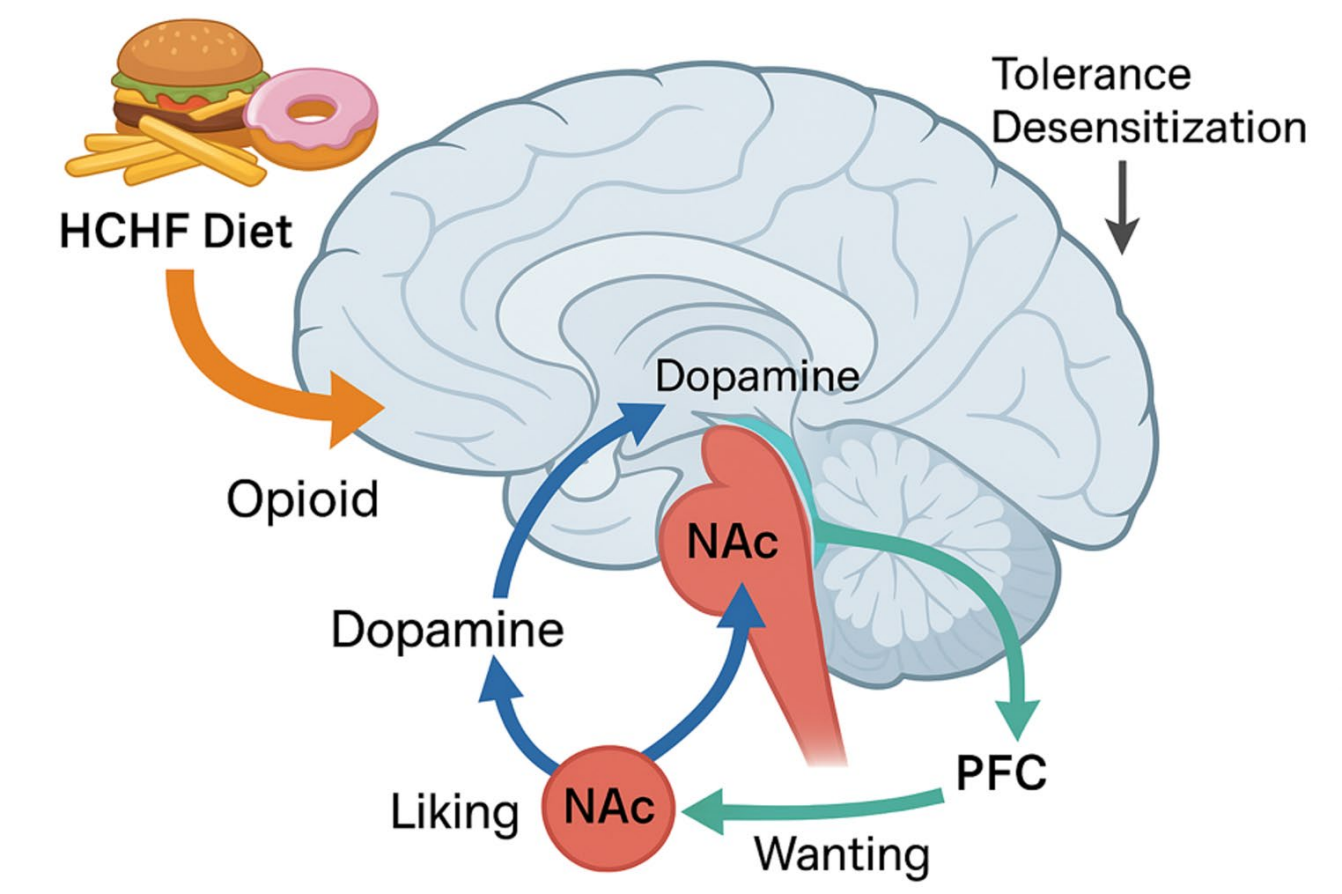
High-Fat High-Sugar Diets and Reward Circuitry

The synchronized interaction of these three structures forms the neurobiological basis of pleasure and reinforcement-based behaviors, not limited to merely meeting physiological needs. An overview of the mesocorticolimbic structures and their interactions is illustrated in Fig. 1.

## Neurobiological Differences between Homeostatic and Hedonic Eating Behaviors

Eating behavior is primarily controlled by two major motivational systems: homeostatic and hedonic. Homeostatic eating is a behavior that develops in response to physiological hunger to maintain the organism’s energy and nutritional balance. This system is regulated by energy-sensing mechanisms in the hypothalamus and operates when combined with metabolic signals such as leptin and ghrelin [15, 16]. On the contrary, hedonic eating is characterized by the continuous consumption of highly rewarding foods for pleasure, even after calorie requirements have been satisfied [17]. This behavior is mainly independent of homeostatic cues and is primarily regulated by the mesolimbic dopamine system [18]. Foods heavy in fat and sugar activate this hedonic process by increasing dopamine release above physiological limitations [19].

Extended overstimulation induces neuroadaptive alterations in the dopaminergic system. Regular consumption of high-calorie, high-fat diets reduce dopamine signaling, resulting in diminished motivational responses to rewards, disruptions in synaptic transmission, and lower expression of D2 receptors [20]. There is a significant overlap between these adaptations and the observed neurological changes. These adaptations largely overlap with the neurobiological changes observed in the addiction process and suggest that excessive eating behavior can be framed within a pathological context.

Research on animals provides an appropriate model for understanding the impact of HFHS diets on the brain’s reward system. Experimental studies, especially in rodents, have shown that these diets can profoundly alter both the structure and function of the dopaminergic system [21–23]. A study by Johnson and Kenny (2010) indicated that rats consuming a diet rich in fat and sugar exhibited markedly reduced dopamine D2 receptor expression. With fewer receptors available, stronger stimulation is needed to reach the same level of reward, leading to the development of tolerance. The findings additionally showed that these rats exhibited decreased interest in natural rewards during reward motivation assessments, however displayed increased sensitivity to pharmaceutical stimulants like cocaine. The results suggest that HFHS diets affect both feeding behavior and the propensity for establishing addicted behaviors [20]. More recent research supports this by showing that long-term exposure to high-fat diets changes synaptic transmission in the NAc, which interferes with the regulation of dopamine release in adolescent rats [23]. These diets impact both the structural and functional integrity of the reward system, as demonstrated by Han et al.’s (2021) findings that young mice fed an HFHS diet showed dopaminergic changes, behavioral abnormalities, and weight gain [22]. Consistent, uncontrolled consumption of high-calorie foods causes neuroplastic changes in the reward system like those produced by addictive substances, increasing susceptibility to these foods on both behavioral and neurobiological levels. This provides support for the argument that food addiction shares neurobiological features with substance addictions, but more research is required before drawing definitive conclusions.

### Dopamine-Opioid Interaction

Although the brain’s dopaminergic and opioid pathways operate independently, they work together within an interconnected network that brings together the motivational (‘wanting’) and hedonic (‘liking’) aspects of reward [18]. The neurochemical interaction between these two systems is significant for explaining, at the neurobiological level, the persistent and compulsive consumption of stimuli with high reward value such as foods rich in sugar and fat [13].

The brain’s endogenous opioid system, primarily through µ-opioid receptors, plays a crucial role in regulating our pleasure and enjoyment (liking). More than that, it acts as a higher-level control mechanism that indirectly modulates the dopamine system. This interaction is most clearly seen in the VTA. Normally, the activity of dopamine neurons in the VTA is kept in check by inhibitory GABAergic interneurons. However, opioids put a brake on these GABAergic neurons [24]. By doing so, they lift the inhibition on dopamine neurons, which then leads to an increase in dopamine release into the NAc through a process called disinhibition [25]. This neurophysiological mechanism highlights the pro-motor effect of the opioid system, showing how it boosts dopaminergic activity.

This mutual interaction creates a feedback loop that feeds the reinforcement cycle in both directions. While the dopamine system sustains our motivational drive (or wanting) for food, the opioid system enhances the pleasure we get from eating, which in turn strengthens the behavior [26]. For this reason, foods that are HFHS trigger a synergistic activation of these two systems because of their both pleasurable and desirable qualities.

Ultimately, the dopamine-opioid interaction is a critical mechanism that explains not only the holistic function of the reward system but also the creation of neurobiological conditions that can lead to food addiction. Over time, this cycle can trigger pathological processes associated with addiction, such as desensitization and the development of compulsive behaviors. Therefore, a closer examination of the interaction between these two neurotransmitter systems is a significant area of research that can contribute to both our understanding of eating disorders and the development of new treatment strategies.

## Effects of High-Fat and High-Sugar Diets on the Dopamine System

### Effects of Acute Consumption

Foods high in fat and sugars strongly activate the dopamine system, particularly after acute consumption, creating intense dopaminergic responses in the brain’s reward system [27, 28]. This process, much like with psychoactive substances, generates a “reward surge” that boosts reward anticipation and motivational drive.

Evidence from both animal models and human fMRI studies shows that the combination of sugar and fat leads to a significantly stronger dopamine release in the striatum compared to sugar or fat alone [29–32]. This synergistic effect triggers dopamine release from the VTA to the NAc, causing the individual to exhibit an increased drive and a tendency to consume the food again [29].

Especially in obesogenic environments, as the frequency of exposure to such foods increases, an individual’s reward sensitivity is further stimulated. This, on a behavioral level, sets the stage for more frequent and automatic eating behaviors. Human neuroimaging studies have shown that exposure to images of fast food or sweet treats increases dopamine-related activation in the NAc and orbitofrontal cortex, and this activation correlates with subjective “craving” scores [33].

### Effects of Chronic Consumption

Prolonged consumption of HFHS diets leads to a series of maladaptive neuroplastic changes within the dopaminergic system, causing both structural and functional impairments. This process is commonly described in addiction literature by the concepts of “reward hyposensitivity” and “incentive disengagement” [19].

Research with animals consistently shows that extended exposure to high-fat, high-sugar diets affect dopaminergic transmission, particularly through decreased availability of D2 receptors and modified reward sensitivity. Han and colleagues (2021) indicated that mice exposed to a high-fat, high-sugar diet developed behavioral deficits with significant changes in dopamine function, implying that prolonged consumption compromises both the structural and functional integrity of the reward system [22]. Fritz et al. (2017) noted that mice consuming a Western-style high-fat, high-sugar diet had modifications in striatal dopamine transmission, supporting the idea that sustained intake results in reduced reward response and tolerance-like adaptations [30].

Furthermore, chronic HFHS consumption is believed to weaken both the tonic and phasic components of dopamine release. This could lead individuals to exhibit a blunted response to environmental rewards and even show signs of hedonic fatigue (anhedonia) [32]. In this context, the dopamine system’s persistent “overload” from constant high stimulation eventually causes the system to desensitize and its motivational capacity to decline. These adaptive impairments strengthen the neurobiological evidence that HFHS diets are not merely a source of short-term pleasure but can, over time, create addiction-like alterations in an individual’s reward system.

### Neurobiological Parallels with Substance Addiction

The dopaminergic effects of HFHS diets are frequently compared to those of psychoactive substance addiction in the literature. It is well-established that substances like cocaine, amphetamine, and nicotine target the dopamine system, causing an immediate and intense dopamine release. Interestingly, HFHS foods have been shown to activate similar neurochemical pathways, triggering the same circuits within the mesolimbic dopamine system [4].

For example, a study by Winterdahl et al. (2019) reported that sugar water consumption in rats, like cocaine, reduced dopamine D2/3 receptor binding capacity and affected µ-opioid receptor activity [34]. Such findings suggest that food consumption leaves traces that overlap with addiction, not just on a behavioral but also on a neurochemical level. This parallel supports the “food addiction” hypothesis, which suggests that compulsive eating disorders and obesity are maintained not only by psychological or environmental factors but also by mechanisms like addiction on a neurobiological level. Consequently, these similarities have opened a research area focused on adapting pharmacological or behavioral treatments known to be effective for substance use disorders to the treatment of food addiction.

### Effects of HFHS Diets on the Endogenous Opioid System

The endogenous opioid system is a neurotransmitter system that regulates pleasure and motivational processes through three main receptor types: µ (mu), δ (delta), and κ (kappa). The key components of this system endorphins, enkephalins, and dynorphins play an active role in both emotional and physiological reward processes [35]. The opioid system, in contrast to the dopamine system, is largely linked to the hedonic (pleasure-giving) component of a reward (liking), which establishes the person’s subjective degree of satisfaction from the interaction [36]. The main structures influenced by this system include the nucleus accumbens (especially its shell region), the amygdala, the insula, and the orbitofrontal cortex. Opioid activation in these areas intensifies significantly during the consumption of highly palatable foods. This moves a person’s relationship with food beyond a simple homeostatic need, giving it an emotional and reinforcing dimension [35].

Foods high in fat and sugars have a strong neurochemical impact on the opioid and dopaminergic systems. Eating foods high in HFHS causes an increase in µ-opioid receptor activation, which can result in a pleasurable experience as well as momentary emotional regulation [37]. The neurobehavioral manifestation of this is often called “comfort eating” in the literature [38]. A person may turn to high-calorie foods to suppress or regulate emotions such as stress, anxiety, or emotional fluctuations, even when they are physiologically full [39]. This behavioral pattern may also be linked to the opioid system’s buffering effect against stress. Animal studies have shown that subjects under stress are more likely to seek out high-fat foods, and this consumption increases endorphin levels in the brain [40]. Furthermore, human studies support these findings. fMRI-based research has observed an increase in µ-opioid receptor activation in pleasure-related brain regions like the insula and orbitofrontal cortex during HFHS food consumption [41]. According to Ziauddeen and Fletcher (2013), this activation implies that eating behavior is sustained not only on a metabolic level but also on an emotional and addiction-like level [42]. The endogenous opioid system has been one of the primary targets of pharmacological intervention. The µ-opioid receptor antagonist naltrexone reduces cravings for calorie-dense foods, according to studies on both humans and animals. In animal models, naltrexone treatment dramatically reduced the intake of high-fat foods and blocked opioid signaling in areas of the brain linked to rewards [18, 26]. Naltrexone has been shown in human studies to lessen food cravings and impulsive eating behaviors, particularly in people with a history of eating disorders [43]. Nevertheless, naltrexone’s effects can differ from person to person, and a person’s reaction to this treatment can be influenced by genetic factors, particularly polymorphisms in the OPRM1 gene [44]. This emphasizes how crucial tailored treatment strategies will be in the future.

## Neuroplasticity and Behavioral Changes

Neuroplasticity is the process of permanent neural reorganization where synaptic connections strengthen or weaken in response to new experiences or environmental stimuli. This process forms the neurobiological basis for cognitive functions like learning and memory [45]. Food-related cues such as taste, smell, and visual signals can be transformed into automatic behavioral patterns through reinforcement learning in the reward system.

Specifically, with repeated consumption of HFHS foods, stimulus-sensitivity increases within the networks connecting the nucleus accumbens, amygdala, and orbitofrontal cortex. This process causes cues associated with a reward (like seeing a dessert in a store or a hamburger in an ad) to trigger dopaminergic activation. This means an individual may engage in eating behavior even without experiencing physiological hunger, which results in the eating behavior moving beyond cognitive control [46].

This cue-based drive is defined in addiction literature as “cue-induced craving” and is a neural mechanism that directly overlaps with substance use disorders [47]. Human fMRI studies have shown that activation of the anterior cingulate cortex (ACC), insula, and NAc in obese individuals is significantly higher in response to food images compared to healthy individuals [48]. This indicates that neuroplastic changes modulate not only biological hunger but also the automatic responses given to environmental cues.

It has been shown that HFHS diets affect not only synaptic transmission but also brain volume, gray matter density, and structural integrity. These changes are concentrated in the PFC, anterior cingulate cortex, and insula, which are areas involved in the reward system and executive function [49]. In animal models, participants on a diet rich in fat and sugar had reduced levels of Brain-Derived Neurotrophic Factor (BDNF) in the frontal cortex and hippocampus. This decline was shown to negatively impact learning performance and synaptic plasticity [50]. Additionally, behavioral outcomes like impaired inhibitory control and increased impulsivity are facilitated by the decline in BDNF.

Studies on humans have documented similar structural alterations. Neuroimaging studies of obese individuals have revealed a reduction in gray matter volume, particularly in the orbitofrontal cortex, associated with impairments in cognitive functions like risk assessment, self-regulation, and decision-making [51]. These findings suggest that long-term consumption of HFHS diets structurally affects not just the acute reward system, but also cognitive control and behavioral regulation functions.

When these neuroplastic changes are combined with the automatization of eating behaviors, hypersensitivity to environmental cues, and loss of cognitive control, they can result in clinical-level compulsive eating and food addiction. In this cycle, an individual may eat without feeling hungry, lose control over the amount they eat, develop feelings of regret and withdrawal after consumption, and then be inclined to repeat the same behavior.

This pattern of behavior presents a strong case that it meets most of the addiction criteria defined in the DSM-5, particularly with negative reinforcement where the motivation is driven more by seeking relief and stress regulation than by pleasure [2]. Neuroplasticity is a key factor in the permanence of these behavioral patterns and stands out as a neurobiological process that should be targeted in treatment approaches.

This review has several limitations. As this is a narrative review, the choice of studies may reflect some selection bias, and the findings should therefore be interpreted with caution. Much of the evidence comes from animal research, which provides valuable insights but does not always translate directly to humans. In addition, the studies vary widely in their dietary protocols, genetic models, and environmental conditions, making it difficult to draw direct comparisons. Finally, there is still a lack of long-term, large-scale data to clearly establish causal links between HFHS diets and neurobiological changes.

## Conclusion

This review comprehensively evaluated the effects of HFHS diets on the brain’s reward system, focusing on dopaminergic and opioid mechanisms. The research suggests that these diets significantly activate the mesocorticolimbic system, increasing motivational and hedonic processes that promote habitual intake. Short-term exposure increases dopamine release and pleasure reactions, however long-term use leads to pathological changes including D2 receptor downregulation, synaptic desensitization, neuroplastic modifications, and structural impairments in the prefrontal cortex. These changes collectively reflect the neurological and behavioral characteristics of drug addiction, confirming the perspective that “food addiction” possesses both a clinical and mechanistic basis.

While current literature has made significant progress in understanding the effects of HFHS diets on the reward system, some fundamental questions remain unanswered. Our understanding of how both systems work simultaneously is still limited. The temporal dynamics of dopamine and opioid release during HFHS consumption, how these systems synchronize with each other, and how one system modulates the other need to be investigated in more detail. Genetic variants (e.g., DRD2, OPRM1) and hormonal states (e.g., leptin resistance, cortisol levels) may shape an individual’s neurological response to HFHS diets. Therefore, identifying individual neurobiological sensitivities is crucial, especially for personalized treatment strategies. Whether the neuroplastic changes caused by HFHS diets can be reversed remains a critical unanswered question. Further research is needed to clarify how methods like cognitive behavioral therapies, dopamine modulators, or opioid antagonists affect these changes and whether they can reorganize brain circuits.

## Key References

- Han J, Nepal P, Odelade A, et al. High-Fat Diet-Induced Weight Gain, Behavioral Deficits, and Dopamine Changes in Young C57BL/6J Mice. Front Nutr. 2021;7.
  - ○ This study demonstrates how early exposure to high-fat diets alters dopamine function and behavior in mice, providing evidence for age-related vulnerability to dietary effects.
- Plaza-Briceño W, Velásquez VB, Silva-Olivares F, et al. Chronic Exposure to High Fat Diet Affects the Synaptic Transmission That Regulates the Dopamine Release in the Nucleus Accumbens of Adolescent Rats. Int J Mol Sci. 2023;24(5).
  - ○ This article provides mechanistic evidence of how HFHS diets disrupt dopamine release regulation in adolescent rats, highlighting synaptic-level vulnerabilities linked to compulsive eating.
- McDougle M, de Araujo A, Singh A, et al. Separate gut-brain circuits for fat and sugar reinforcement combine to promote overeating. Cell Metab. 2024;36(2):393- 407.e7.
  - ○ This cutting-edge study identifies distinct gut–brain circuits for fat and sugar reinforcement that act synergistically to promote overeating, advancing understanding of diet–brain interactions.

## Data Availability

No datasets were generated or analysed during the current study.

## References

[1] Monteiro CA, Cannon G, Levy RB, et al. Ultra-processed foods: what they are and how to identify them. Public Health Nutr. 2019;22:936–41. 10.1017/S1368980018003762. 30744710 10.1017/S1368980018003762 PMC10260459

[2] Gearhardt AN, Corbin WR, Brownell KD. Food addiction: an examination of the diagnostic criteria for dependence. J Addict Med. 2009;3:1–7. 10.1097/ADM.0B013E318193C993. 21768996 10.1097/ADM.0b013e318193c993

[3] Mercola J, D’Adamo CR. Linoleic acid: a narrative review of the effects of increased intake in the standard American diet and associations with chronic disease. Nutrients. 2023. 10.3390/NU15143129. 37513547 10.3390/nu15143129 PMC10386285

[4] Volkow ND, Wang GJ, Tomasi D, Baler RD. The addictive dimensionality of obesity. Biol Psychiatry. 2013;73:811–8. 10.1016/j.biopsych.2012.12.020. 23374642 10.1016/j.biopsych.2012.12.020 PMC4827347

[5] Finlayson G. Food addiction and obesity: unnecessary medicalization of hedonic overeating. Nat Rev Endocrinol. 2017;13:493–8. 10.1038/nrendo.2017.61. 28549063 10.1038/nrendo.2017.61

[6] Ziauddeen H, Alonso-Alonso M, Hill JO, et al. Obesity and the neurocognitive basis of food reward and the control of intake. Adv Nutr. 2015;6:474–86. 10.3945/an.115.008268. 26178031 10.3945/an.115.008268 PMC4496739

[7] Schulte EM, Potenza MN, Gearhardt AN. A commentary on the eating addiction versus food addiction perspectives on addictive-like food consumption. Appetite. 2017;115:9–15. 10.1016/j.appet.2016.10.033. 27984189 10.1016/j.appet.2016.10.033

[8] Avena NM, Rada P, Hoebel BG. Evidence for sugar addiction: behavioral and neurochemical effects of intermittent, excessive sugar intake. Neurosci Biobehav Rev. 2008;32:20–39. 10.1016/j.neubiorev.2007.04.019. 17617461 10.1016/j.neubiorev.2007.04.019 PMC2235907

[9] Schulte EM, Avena NM, Gearhardt AN. Which foods may be addictive? The roles of processing, fat content, and glycemic load. PLoS One. 2015. 10.1371/JOURNAL.PONE.0117959. 25692302 10.1371/journal.pone.0117959 PMC4334652

[10] Berridge KC, Robinson TE. Liking, wanting, and the incentive-sensitization theory of addiction. Am Psychol. 2016;71:670–9. 10.1037/AMP0000059. 27977239 10.1037/amp0000059 PMC5171207

[11] Arias-Carrián O, Stamelou M, Murillo-Rodríguez E, et al. Dopaminergic reward system: A short integrative review. Int Arch Med. 2010. 10.1186/1755-7682-3-24. 3:. 10.1186/1755-7682-3-24 PMC2958859 20925949

[12] Ikemoto S, Bonci A. Neurocircuitry of drug reward. Neuropharmacology. 2014;76:329–41. 10.1016/J.NEUROPHARM.2013.04.031. 23664810 10.1016/j.neuropharm.2013.04.031 PMC3772961

[13] Polk SE, Schulte EM, Furman CR, Gearhardt AN. Wanting and liking: separable components in problematic eating behavior? Appetite. 2017;115:45–53. 10.1016/j.appet.2016.11.015. 27840087 10.1016/j.appet.2016.11.015 PMC5796412

[14] Bechara A. Decision making, impulse control and loss of willpower to resist drugs: a neurocognitive perspective. Nat Neurosci. 2005;8:1458–63. 10.1038/NN1584. 16251988 10.1038/nn1584

[15] Uribe-Cerda S, Morselli E, Perez-Leighton C. Updates on the neurobiology of food reward and their relation to the obesogenic environment. Curr Opin Endocrinol Diabetes Obes. 2018;25:292–7. 10.1097/MED.0000000000000427. 30063551 10.1097/MED.0000000000000427

[16] Berthoud HR. The neurobiology of food intake in an obesogenic environment. Proc Nutr Soc. 2012;71:478–87. 10.1017/S0029665112000602. 22800810 10.1017/S0029665112000602 PMC3617987

[17] Campos A, Port JD, Acosta A. Integrative hedonic and homeostatic food intake regulation by the central nervous system: insights from neuroimaging. Brain Sci. 2022. 10.3390/BRAINSCI12040431. 35447963 10.3390/brainsci12040431 PMC9032173

[18] Morales I, Berridge KC. ‘Liking’ and ‘wanting’ in eating and food reward: brain mechanisms and clinical implications. Physiol Behav. 2020. 10.1016/j.physbeh.2020.113152. 32846152 10.1016/j.physbeh.2020.113152 PMC7655589

[19] Volkow ND, Wang GJ, Fowler JS. Food and drug reward: overlapping circuits in human obesity and addiction. Curr Top Behav Neurosci. 2012;11:1–24. 10.1007/7854_2011_169. 22016109 10.1007/7854_2011_169

[20] Johnson PM, Kenny PJ. Dopamine D2 receptors in addiction-like reward dysfunction and compulsive eating in obese rats. Nat Neurosci. 2010;13:635–41. 10.1038/nn.2519. 20348917 10.1038/nn.2519 PMC2947358

[21] Baik JH. Dopamine signaling in food addiction: role of dopamine D2 receptors. BMB Rep. 2013;46:519–26. 10.5483/BMBREP.2013.46.11.207. 24238362 10.5483/BMBRep.2013.46.11.207 PMC4133846

[22] Han J, Nepal P, Odelade A, et al. High-fat diet-induced weight gain, behavioral deficits, and dopamine changes in young C57BL/6J mice. Front Nutr. 2021. 10.3389/FNUT.2020.591161. 33553228 10.3389/fnut.2020.591161 PMC7855171

[23] Plaza-Briceño W, Velásquez VB, Silva-Olivares F, et al. Chronic exposure to high fat diet affects the synaptic transmission that regulates the dopamine release in the nucleus accumbens of adolescent male rats. Int J Mol Sci. 2023. 10.3390/IJMS24054703. 36902133 10.3390/ijms24054703 PMC10003643

[24] Fields HL, Hjelmstad GO, Margolis EB, Nicola SM. Ventral tegmental area neurons in learned appetitive behavior and positive reinforcement. Annu Rev Neurosci. 2007;30:289–316. 10.1146/ANNUREV.NEURO.30.051606.094341. 17376009 10.1146/annurev.neuro.30.051606.094341

[25] Han X, Jing Myi, Zhao Tyun, et al. Role of dopamine projections from ventral tegmental area to nucleus accumbens and medial prefrontal cortex in reinforcement behaviors assessed using optogenetic manipulation. Metab Brain Dis. 2017;32:1491–502. 10.1007/s11011-017-0023-3. 28523568 10.1007/s11011-017-0023-3

[26] Castro DC, Berridge KC. Opioid hedonic hotspot in nucleus accumbens shell: mu, delta, and kappa maps for enhancement of sweetness liking and wanting. J Neurosci. 2014;34:4239–50. 10.1523/JNEUROSCI.4458-13.2014. 24647944 10.1523/JNEUROSCI.4458-13.2014 PMC3960467

[27] Joshi A, Faivre F, la Fleur SE, Barrot M. Midbrain and lateral nucleus accumbens dopamine depletion affects free-choice high-fat high-sugar diet preference in male rats. Neuroscience. 2021;467:171–84. 10.1016/j.neuroscience.2021.05.022. 34048800 10.1016/j.neuroscience.2021.05.022

[28] Fordahl SC, Jones SR. High-fat-diet-induced deficits in dopamine terminal function are reversed by restoring insulin signaling. ACS Chem Neurosci. 2017;8:290–9. 10.1021/ACSCHEMNEURO.6B00308. 27966885 10.1021/acschemneuro.6b00308 PMC5789793

[29] DiFeliceantonio AG, Coppin G, Rigoux L, et al. Supra-additive effects of combining fat and carbohydrate on food reward. Cell Metab. 2018;28:33–44.e3. 10.1016/j.cmet.2018.05.018. 29909968 10.1016/j.cmet.2018.05.018

[30] Fritz BM, Muñoz B, Yin F, et al. A high-fat, high-sugar ‘western’ diet alters dorsal striatal glutamate, opioid, and dopamine transmission in mice. Neuroscience. 2018;372:1–15. 10.1016/j.neuroscience.2017.12.036. 29289718 10.1016/j.neuroscience.2017.12.036 PMC5809281

[31] McDougle M, de Araujo A, Singh A, et al. Separate gut-brain circuits for fat and sugar reinforcement combine to promote overeating. Cell Metab. 2024;36:393–407.e7. 10.1016/j.cmet.2023.12.014. 38242133 10.1016/j.cmet.2023.12.014 PMC11898112

[32] Tellez LA, Medina S, Han W, et al. A gut lipid messenger links excess dietary fat to dopamine deficiency. Science. 2013;341:800–2. 10.1126/SCIENCE.1239275. 23950538 10.1126/science.1239275

[33] Stice E, Spoor S, Bohon C, et al. Relation of reward from food intake and anticipated food intake to obesity: A functional magnetic resonance imaging study. J Abnorm Psychol. 2008;117:924–35. 10.1037/A0013600. 19025237 10.1037/a0013600 PMC2681092

[34] Winterdahl M, Noer O, Orlowski D, et al. Sucrose intake lowers μ-opioid and dopamine D2/3 receptor availability in porcine brain. Sci Rep. 2019. 10.1038/S41598-019-53430-9. 31729425 10.1038/s41598-019-53430-9 PMC6858372

[35] Le Merrer J, Becker JAJ, Befort K, Kieffer BL. Reward processing by the opioid system in the brain. Physiol Rev. 2009;89:1379–412. 10.1152/PHYSREV.00005.2009. 19789384 10.1152/physrev.00005.2009 PMC4482114

[36] Berridge KC, Kringelbach ML. Pleasure systems in the brain. Neuron. 2015;86:646–64. 10.1016/j.neuron.2015.02.018. 25950633 10.1016/j.neuron.2015.02.018 PMC4425246

[37] Gosnell BA, Levine AS. Reward systems and food intake: role of opioids. Int J Obes. 2009;33:S54–8. 10.1038/IJO.2009.73. 10.1038/ijo.2009.73 19528981

[38] Macht M. How emotions affect eating: a five-way model. Appetite. 2008;50:1–11. 10.1016/J.APPET.2007.07.002. 17707947 10.1016/j.appet.2007.07.002

[39] Dallman MF, Pecoraro NC, La Fleur SE. Chronic stress and comfort foods: Self-medication and abdominal obesity. Brain Behav Immun. 2005;19:275–80. 10.1016/j.bbi.2004.11.004. 15944067 10.1016/j.bbi.2004.11.004

[40] Cai H, Haubensak W, Anthony TE, Anderson DJ. Central amygdala PKC-δ + neurons mediate the influence of multiple anorexigenic signals. Nat Neurosci. 2014;17:1240–8. 10.1038/NN.3767. 25064852 10.1038/nn.3767 PMC4146747

[41] Nummenmaa L, Saanijoki T, Tuominen L, et al. μ-opioid receptor system mediates reward processing in humans. Nat Commun. 2018. 10.1038/S41467-018-03848-Y. 29662095 10.1038/s41467-018-03848-y PMC5902580

[42] Ziauddeen H, Fletcher PC. Is food addiction a valid and useful concept? Obes Rev. 2013;14:19–28. 10.1111/J.1467-789X.2012.01046.X. 23057499 10.1111/j.1467-789X.2012.01046.x PMC3561707

[43] Ziauddeen H, Farooqi IS, Fletcher PC. Obesity and the brain: how convincing is the addiction model? Nat Rev Neurosci. 2012;13:279–86. 10.1038/NRN3212. 22414944 10.1038/nrn3212

[44] Anton RF, Oroszi G, O’Malley S, et al. An evaluation of µ-opioid receptor (OPRM1) as a predictor of naltrexone response in the treatment of alcohol dependence: results from the combined pharmacotherapies and behavioral interventions for alcohol dependence (COMBINE) study. Arch Gen Psychiatry. 2008;65:135–44. 10.1001/ARCHPSYC.65.2.135. 18250251 10.1001/archpsyc.65.2.135 PMC2666924

[45] Kolb B, Whishaw IQ. Brain plasticity and behavior. Annu Rev Psychol. 1998;49:43–64. 10.1146/ANNUREV.PSYCH.49.1.43. 9496621 10.1146/annurev.psych.49.1.43

[46] Stoeckel LE, Weller RE, Cook EW, et al. Widespread reward-system activation in obese women in response to pictures of high-calorie foods. Neuroimage. 2008;41:636–47. 10.1016/j.neuroimage.2008.02.031. 18413289 10.1016/j.neuroimage.2008.02.031

[47] Volkow ND, Wang GJ, Fowler JS, et al. Addiction: beyond dopamine reward circuitry. Proc Natl Acad Sci U S A. 2011;108:15037–42. 10.1073/PNAS.1010654108. 21402948 10.1073/pnas.1010654108 PMC3174598

[48] Brooks SJ, Cedernaes J, Schiöth HB. Increased prefrontal and parahippocampal activation with reduced dorsolateral prefrontal and insular cortex activation to food images in obesity: a meta-analysis of fMRI studies. PLoS One. 2013. 10.1371/JOURNAL.PONE.0060393. 23593210 10.1371/journal.pone.0060393 PMC3622693

[49] Kanoski SE, Davidson TL. Western diet consumption and cognitive impairment: links to hippocampal dysfunction and obesity. Physiol Behav. 2011;103:59–68. 10.1016/J.PHYSBEH.2010.12.003. 21167850 10.1016/j.physbeh.2010.12.003 PMC3056912

[50] Molteni R, Barnard RJ, Ying Z, et al. A high-fat, refined sugar diet reduces hippocampal brain-derived neurotrophic factor, neuronal plasticity, and learning. Neuroscience. 2002;112:803–14. 10.1016/S0306-4522(02)00123-9. 12088740 10.1016/s0306-4522(02)00123-9

[51] Carnell S, Gibson C, Benson L, et al. Neuroimaging and obesity: current knowledge and future directions. Obes Rev. 2012;13:43–56. 21902800 10.1111/j.1467-789X.2011.00927.x PMC3241905

